# HeartCV: a tool for transferrable, automated measurement of heart rate and heart rate variability in transparent animals

**DOI:** 10.1242/jeb.244729

**Published:** 2022-10-03

**Authors:** Ziad Ibbini, John I. Spicer, Manuela Truebano, John Bishop, Oliver Tills

**Affiliations:** ^1^ Marine Biology and Ecology Research Centre, Plymouth University, Plymouth PL4 8AA, UK; ^2^ Marine Biological Association of the UK, Citadel Hill Laboratory, Plymouth PL1 2PB, UK

**Keywords:** Heat rate variability, Ecophysiology, Cardiac activity, Software, Image-analysis

## Abstract

Heart function is a key component of whole-organismal physiology. Bioimaging is commonly, but not exclusively, used for quantifying heart function in transparent individuals, including early developmental stages of aquatic animals, many of which are transparent. However, a central limitation of many imaging-related methods is the lack of transferability between species, life-history stages and experimental approaches. Furthermore, locating the heart in mobile individuals remains challenging. Here, we present HeartCV: an open-source Python package for automated measurement of heart rate and heart rate variability that integrates automated localization and is transferrable across a wide range of species. We demonstrate the efficacy of HeartCV by comparing its outputs with measurements made manually for a number of very different species with contrasting heart morphologies. Lastly, we demonstrate the applicability of the software to different experimental approaches and to different dataset types, such as those corresponding to longitudinal studies.

## Teaser

**Summary:** HeartCV is an open-source Python package that can automatically measure cardiac rhythm traits in a wide range of species, life history stages and experimental designs.

## Introduction

Heart rate (HR) and heart rate variability (HRV) are key measures of heart function (Stein et al., 1994; Malik et al., 1996; Acharya et al., 2006) and in many species are proven indicators of cardiovascular health, susceptibility to heart disease and cardiac arrest (Stein et al., 1994; Malik et al., 1996; Norman et al., 2012; Zena et al., 2021). HR and HRV can be measured using a broad range of approaches, including ultrasound, electrocardiography and bioimaging (see Santoso et al., 2020a; Zabihihesari et al., 2021). Bioimaging is a common approach for species or developmental stages with a transparent body wall (Brainerd and Hale, 2006; Salman and Yalcin, 2020; Burggren, 2021) and is often preferred over other approaches because of its transferability between species and life-history stages (Colmorgen and Paul, 1995; Swedlow et al., 2009; Meijering et al., 2016). Bioimaging is not reliant on a particular experimental setup (Colmorgen and Paul, 1995; Schindelin et al., 2012) and is non-invasive unlike other acquisition technologies, such as electrocardiography (Colmorgen and Paul, 1995; Zabihihesari et al., 2021). Furthermore, advancements in imaging technologies have improved both the quality and throughput of image acquisitions (Shariff et al., 2010; Walter et al., 2010; Swedlow et al., 2009; Salman and Yalcin, 2020). However, the size and magnitude of the image datasets generated using these technologies can extend to millions of images and hundreds of samples (Swedlow et al., 2009; Meijering et al., 2016; Tills et al., 2018). Subsequent manual image analysis presents significant data processing bottlenecks and can be limited by the repeatability of analysis and human error (Zhou and Wong, 2006; Cardona and Tomancak, 2012). Thus, the development of automated computer vision methods to address these issues is an important endeavor, especially within the broader field of bioimage informatics (Swedlow et al., 2009; Cardona and Tomancak, 2012; Myers, 2012).

Many researchers have developed semi- and fully-automated computer vision methods to quantify both HR and HRV (Chan et al., 2009; Fink et al., 2009; Tills et al., 2018; Gierten et al., 2020; Zabihihesari et al., 2021). Generally, these methods are reliant on relatively simple image measures to identify when heart beats occur, such as mean or standard deviation in pixel values (e.g. Chan et al., 2009; Fink et al., 2009; Tills et al., 2018; Gierten et al., 2020), measures of motion revealed via frame subtraction approaches (e.g. Fink et al., 2009; Zabihihesari et al., 2021) or M-modes, often termed dynamic kymographs (e.g. Fink et al., 2009; Kurnia et al., 2021). These image measures can be derived from any video produced via bioimaging, and therefore should be applicable to any species or life-history stage with visible cardiac activity. However, accurate results typically depend on a localization step to identify the region containing the heart, enabling the removal of non-cardiac noise, such as whole-body movement, to generate an informative cardiac signal. Manual localization is used by a number of techniques (Santoso et al., 2020b; Kurnia et al., 2021), but this greatly limits the size and scale of datasets that can be analyzed. Some methods instead omit localization entirely, requiring sample preparation to limit non-cardiac noise, often via anesthetization, immobilization and/or dissection (Chan et al., 2009; Fink et al., 2009; Hoage et al., 2012; Yozzo et al., 2013; Santoso et al., 2020b). Such sample preparation is time consuming, often invasive and non-reversible, and generally limits applications of the method to tested species and/or specific experimental designs. A number of methods of automated localization have been developed to overcome these issues (e.g. Spomer et al., 2012; De Luca et al., 2014; Pylatiuk et al., 2014; Gierten et al., 2020; Zabihihesari et al., 2021), but are often species or life-history-stage specific, with transferrable methods remaining scarce.

Recently developed techniques that integrate power spectral analysis into software-driven localization have proven effective at measuring both complex integrative physiological signals and heart rate (Tills et al., 2018; Gierten et al., 2020). A key advantage of such spectral methods is their transferability between species and life history stages with considerable non-cardiac motion such as muscular contractions and rotational movements (Tills et al., 2018). Cardiac activity is located on the basis that the rhythmic contractions of a heart produce quasi-periodic fluctuations in pixel values between images in videos. Applying power spectral analysis to these fluctuations in pixel values enables identification of the temporal frequencies at which cardiac activity is occurring, and this can therefore be used to identify regions of interest based on the likelihood that they contain cardiac activity (Tills et al., 2018; Gierten et al., 2020). The potential for spectral techniques to support a method capable of capturing both HR and HRV across species, life-history stages and experimental approaches remains untested.

Here, we present HeartCV***,*** an open-source Python package for measurement of HR and HRV that incorporates a transferrable method for automated localization of cardiac regions. We demonstrate the efficacy of HeartCV by comparison with manual measures for disparate aquatic species with radically different heart morphologies: (1) an ascidian (sea squirt), *Ciona intestinalis*; (2) a mollusc (snail), *Radix balthica*; and (3) a crustacean (prawn), *Palaemon serratus*. *Radix balthica* and *P. serratus* possess superficially globular hearts (Smirthwaite et al., 2007; Dantzler, 1997), whereas *C. intestinalis* has a tubular heart (Davidson, 2007), enabling the testing of automated localization of cardiac signals without reliance on a particular cardiac morphology. To evaluate the ability of HeartCV to measure HR and HRV responses in different experimental contexts, we used: (1) ramping thermal assays in *C. intestinalis*; (2) static thermal assays in hippo stage *R. balthica* embryos; and (3) chronically elevated temperatures at different developmental stages of *P. serratus*.

## MATERIALS AND METHODS

### Overview of HeartCV software

**Fig. 1.**
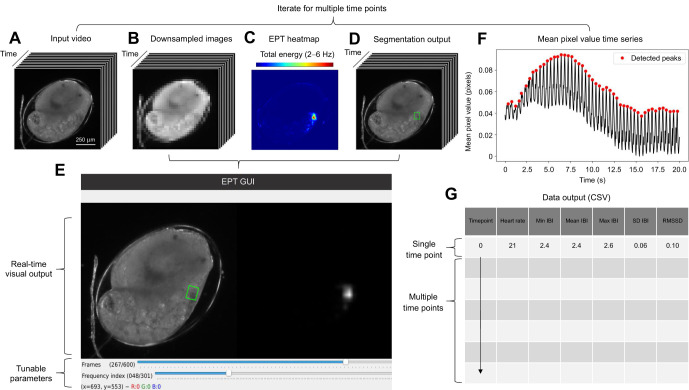
**HeartCV processing pipeline, shown for hippo stage *Radix balthica*.** Video (A) is down-sampled (B) and used to produce energy proxy traits (EPTs; Tills et al., 2021) then visualized as an EPT heatmap (C), where the cardiac region detected via segmentation (D) is used to filter the video and produce an mean pixel value (MPV) signal (F). Steps shown in B–D can be managed via a graphical user interface (GUI), enabling users to scroll through images in the video (upper trackbar) and tune the temporal frequencies (lower trackbar) used to create the EPT heatmap (E). Dynamic visual output is provided comprising segmentation output (left image in GUI) and the EPT heatmap (right image in GUI) (E). Finally, cardiac traits are computed and exported to CSV file (G). Min, minimum; Max, maximum; SD, standard deviation; RMSSD, root mean square of successive differences; IBI, inter-beat interval. Note that steps shown in A–E can be iterated for scenarios involving multiple time points.

HeartCV is a Python package (https://github.com/EmbryoPhenomics/heartcv/) for measuring HR and HRV, via inter-beat intervals from videos of transparent animals. The software operates using a two-stage workflow involving: (i) localization to identify the predominant cardiac region in a video and thus extract a mean pixel value (MPV) signal with minimal non-cardiac noise (Fig. 1A–F), and (ii) peak detection to extract heart beats from this MPV signal (Fig. 1F). Subsequently, HR and inter-beat interval (IBI; the timing between individual heart beats) measures are quantified from the MPV signal (Fig. 1G). HeartCV can be used in experiments involving either single or multiple time points (see https://github.com/EmbryoPhenomics/heartcv/blob/main/examples/example_1.py and https://github.com/EmbryoPhenomics/heartcv/blob/main/examples/example_2.py, respectively). Please refer to the documentation for a detailed guide on installation and usage: https://heartcv.readthedocs.io/en/stable/.

### Localization

HeartCV initially resizes each image in a given video by a user-defined factor to reduce computational load (Fig. 1B). Power spectral analysis is then performed on the resized video to create energy proxy traits (EPTs): the amount of energy within different temporal frequencies in the pixel value fluctuations from video of live biological material (Tills et al., 2018, 2021). EPTs are then filtered to only specific, or a range of, temporal frequencies at which cardiac activity is expected. Filtered EPTs are subsequently collapsed into a two-dimensional heatmap by computing the total energy at these specified temporal frequencies (Fig. 1C). This heatmap is then processed using the OTSU thresholding algorithm (Otsu, 1979), and the largest shape found in the thresholded image using contour detection and filtering is identified as corresponding to the largest region of cardiac activity in the video (Fig. 1D). This cardiac region is isolated using a rectangular bounding box and is used in all subsequent steps. Where necessary, or preferred, the steps of localizing heart function (Fig. 1B–D) can be supervised through the use of a graphical user interface, to finely tune parameters to identify the temporal frequencies corresponding to cardiac activity (Fig. 1E). Note that users can only select specific frequencies via the graphical user interface (e.g. 2.6 Hz), whereas a range can be selected when addressing the underlying functions directly in Python. The final step of the localization stage of HeartCV is the calculation of an MPV time series from the filtered image sequence to enable identification of the timing of individual heat beats (Fig. 1F).

### Peak detection

The MPV time series produced in the localization stage is first up-sampled via linear interpolation to emphasize the dominance of peaks and thereby increase the ability of subsequent analyses to identify the timing of individual heart beats. Automatic multiscale peak detection (AMPD; Scholkmann et al., 2012) is then applied to the processed MPV signal to extract peaks that correspond to heart beats (Fig. 1F). These peaks are directly used to quantify beats per minute (bpm) and IBI measures.

### Experimental approach

#### Ciona intestinalis

Adult individual *Ciona intestinalis* (Linnaeus 1767) were collected by hand from Sutton Harbour Marina in Plymouth, UK (50.22N, 4.08W). They were used to establish a laboratory culture using the methods of Sato et al. (2014) via *in vitro* fertilization. Juveniles were maintained in 5 liter aquaria containing native sea water (NSW) at 15°C with a salinity (*S*) of 34, and were later transferred to larger aquaria (7 liters) at approximately 10 weeks post-fertilization. Full water changes were carried out weekly and individuals were fed *Isochrysis galbana* and *Chaetoceros gracilis ad libitum*.

Initially, 1st ascidian stage juveniles (*n*=13, ∼2 weeks post-fertilization) were imaged (720×540 pixels, 25 frames s^−1^, 8-bit depth) to produce videos (duration=10 s) to validate against manual measures. Imaging was carried out using a Sony Nex-5N camera (Sony, Tokyo, Japan) attached to a Euromex RZ microscope. These videos were then processed using a supervised approach (Fig. 1E; see https://github.com/EmbryoPhenomics/heartcv/blob/main/examples/example_1.py) to produce the validation dataset. To address a hardware issue with the camera resulting in frame doubling that effectively halved the number of usable images, we smoothed the MPV signals produced by HeartCV using the LOWESS algorithm with fraction set to 0.015 (Cleveland, 1979).

To assess the ability of HeartCV to quantify responses to a rapid ramping thermal challenge, 2nd ascidian stage juveniles (*n*=8, ∼8 months post-fertilization) were imaged (QImaging R3 Retiga camera, QImaging, Birmingham, UK: 480×364 pixels, 20 frames s^−1^, 16-bit depth, 10× magnification, attached to a Leica M205C microscope, Leica, Wetzlar, Germany) continuously throughout ramping assays (30 min at a ramping rate of 0.4°C min^−1^ from 16°C to 29°C). Individuals were transferred from aquaria to Petri dishes containing NSW and placed on a heated glass table (T-Glass controlled via a OkoLab H401-T, OkoLab, Naples, Italy) positioned over a dissecting microscope with darkfield lighting (Leica CLS 150 LED). Temperature was monitored using a thermocouple secured in the Petri dish containing the individual and this was logged to the OkoLab H401-T controller. MicroManager (Edelstein et al., 2010) was used to acquire continuous video in the form of OME TIFF stacks that were converted to 8-bit video to be compatible with HeartCV using a custom Python script (https://github.com/EmbryoPhenomics/heartcv/blob/main/utils/conversion_16bit_to_8bit.py). HR and IBI measures were then quantified using an iterative supervised approach, where each video was processed at intervals of 20 s (Fig. 1; https://github.com/EmbryoPhenomics/heartcv/blob/main/examples/example_2.py). An iterative approach with intervals was required here because the cardiac frequencies changed during the exposure trials, and so processing the footage in small intervals was required for accurate measurement of HR and IBI measures.

#### Radix balthica

Embryos of *Radix balthica* (Linnaeus 1758) (*n*=144) were imaged (750×750 pixels, 20 frames s^−1^, 16-bit depth, 200× magnification, subsequently converted to 8-bit depth using a custom Python script: https://github.com/EmbryoPhenomics/heartcv/blob/main/utils/conversion_16bit_to_8bit.py) at chronic temperatures of 20**°**C (*n*=42), 25**°**C (*n*=44) and 30**°**C (*n*=32), for 30 s every hour from the first cell division until hatching. This was achieved using the OpenVIM system: open-source software controlled videomicroscope with a robotic *X–Y* stage for high-throughput time-lapse imaging of developing embryos (see Tills et al., 2018). Embryos were held in 96-well microtiter plates housed within jacket incubation chambers maintained at 20**°**C, 25**°**C or 30**°**C, located within each OpenVIM. This video dataset was subsampled to extract only videos of hippo stage embryos [20**°**C (*n*=10), 25**°**C (*n*=10) and 30**°**C (*n*=10)]. HR and IBI measures were then quantified using the supervised approach to produce the experimental dataset (Fig. 1E). One-way ANOVA followed by Tukey's *post hoc* comparisons was used to test for differences in treatment responses for each of the cardiac measures produced via HeartCV. This was conducted in R v.3.6.3 (https://www.r-project.org/).

Finally, videos (*n*=28) from this subsample were trimmed to the first 10 s for manual validation. Two replicates from the 30°C treatment were excluded because whilst usable MPV signals could be produced via HeartCV, their heart rate was too high to perform reliable manual measurement at the frame rate at which the videos were captured. Capturing video at higher frame rates would help overcome this issue and so we recommend that users capture video at a frame rate at which the cardiac cycle can be observed clearly.

#### Palaemon serratus

Three gravid *Palaemon serratus* (Pennant 1777) females were collected using a hand-held net from tidepools at Jennycliff Bay in Plymouth, UK (50.20N, 4.07W). They were maintained in a 20 liter outdoor tank (*S*=30–40) for 9 days before being transferred to laboratory conditions, where they were kept at ∼15**°**C and salinity of ∼35. Individuals were fed marine pellet (New Era Aquaculture™) *ad libitum* throughout, and supplemented with locally sourced seaweeds in the laboratory (*Fucus serratus*, *Ulva lactuca*, *Chondrus crispus*, *Mastocarpus stellatus*, *Ceramium* spp. and *Cladophora* spp.). Water changes were carried out every other day.

Embryos at each of three different developmental stages were carefully removed from the females' swimmerets when and as required. Developmental stages were defined as follows: ‘Early’: Stage 5, ‘Middle’: Stage 6, ‘Late’: Stage 7, in accordance with Müller et al. (2004). Embryos from each developmental stage were acclimated to 15**°**C throughout development until the treatments commenced. Time-lapse recordings of the three developmental stages of *P. serratus* were acquired (750×750 pixels, 25 frames s^−1^, 16-bit depth, 200x magnification, subsequently converted to 8-bit depth using a custom Python script: https://github.com/EmbryoPhenomics/heartcv/blob/main/utils/conversion_16bit_to_8bit.py) using the OpenVIM system (Tills et al., 2018) at chronic temperatures of 15**°**C (*n*=8 per developmental stage) and 20**°**C (*n*=8 per developmental stage), for 24 s every hour for 40 h. Embryos were held in 96-well microtiter plates housed within jacket incubation chambers maintained at 15**°**C or 20**°**C, located within each OpenVIM. Heart rate and measures of IBI were then quantified using HeartCV through an automated approach for multiple time points (Fig. 1; https://github.com/EmbryoPhenomics/heartcv/blob/main/examples/example_2.py), where the upper and lower frequency limits for filtering EPTs were 2 and 6 Hz, respectively. Note that for time points where automated localization was unsuccessful because of little to no cardiac activity (i.e.<2 heartbeats per time point), cardiac measures were set to empty values (i.e. NAN). Finally, for manual validation, videos (*n*=24, *n*=8 per developmental stage, length=10 s, timepoint=1) from time-lapse recordings corresponding to 15**°**C were subsequently used for quantification of HR and IBI measures manually.

### Manual validation

HR and IBI measures from HeartCV were validated by comparison with measures made via manual observation of the same videos. Manual analysis was performed using a custom web application developed in Python primarily using Dash (v.1.9.1), Flask (v.1.1.2, Grinberg, 2018) and OpenCV (v.4.5.2, Bradski, 2000) (Fig. S1). The application was used to manually record the image(s) at which heart beats occur for a given video. For the globular hearts of *P. serratus* (*n*=24) and *R. balthica* (*n*=28), heartbeats were recorded at the point of end diastole in the cardiac cycle. Conversely, for the tubular heart of *C. intestinalis* (*n*=13), heartbeats were recorded at the point when a contractile wave had completed. For each sample, we validated 10 s of footage against the equivalent measures produced by HeartCV. Formal comparison between manual and automated measures of cardiac traits was carried out using Pearson's correlation coefficient via SciPy (Virtanen et al., 2020).

## RESULTS AND DISCUSSION

### Validation

**Fig. 2.**
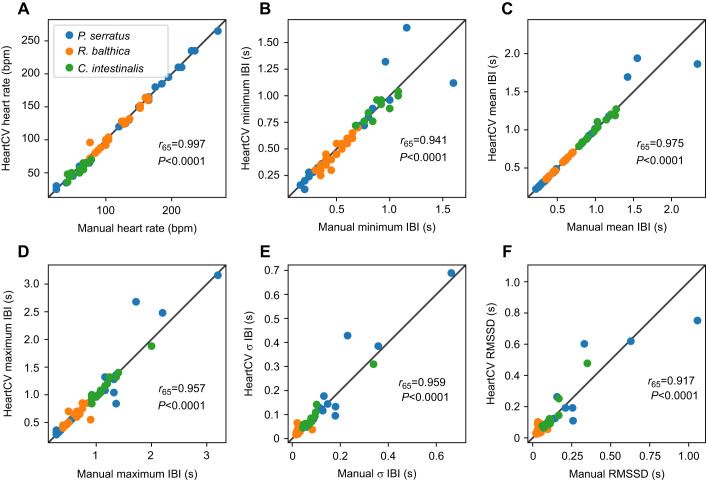
**Comparison of heart rate and inter-beat interval measures quantified using manual counting and HeartCV for the three species studied.** Heart rate (A) and inter-beat interval (IBI) measures (B–F) for *C. intestinalis* (no. juveniles=13), *R. balthica* (no. embryos=30) and *P. serratus* (no. embryos=24). Pearson's correlation coefficient and the accompanying *P*-values are shown in the bottom right of each plot.

Concordance between manual and HeartCV produced data was high for all cardiac measures in all three species tested, despite their very different cardiac morphologies (Fig. 2). The localization workflow was effective without restrictive sample preparation, such as immobilization or dissection, meaning that HeartCV has promising applicability to experimental contexts in which movement cannot be restricted. The method does rely on direct visibility of cardiac function and hence this is a general limitation of the approach, but it is likely to be a less frequent constraint in early developmental stages.

### Cardiac responses to a rapid thermal challenge in *C. intestinalis*

**Fig. 3.**
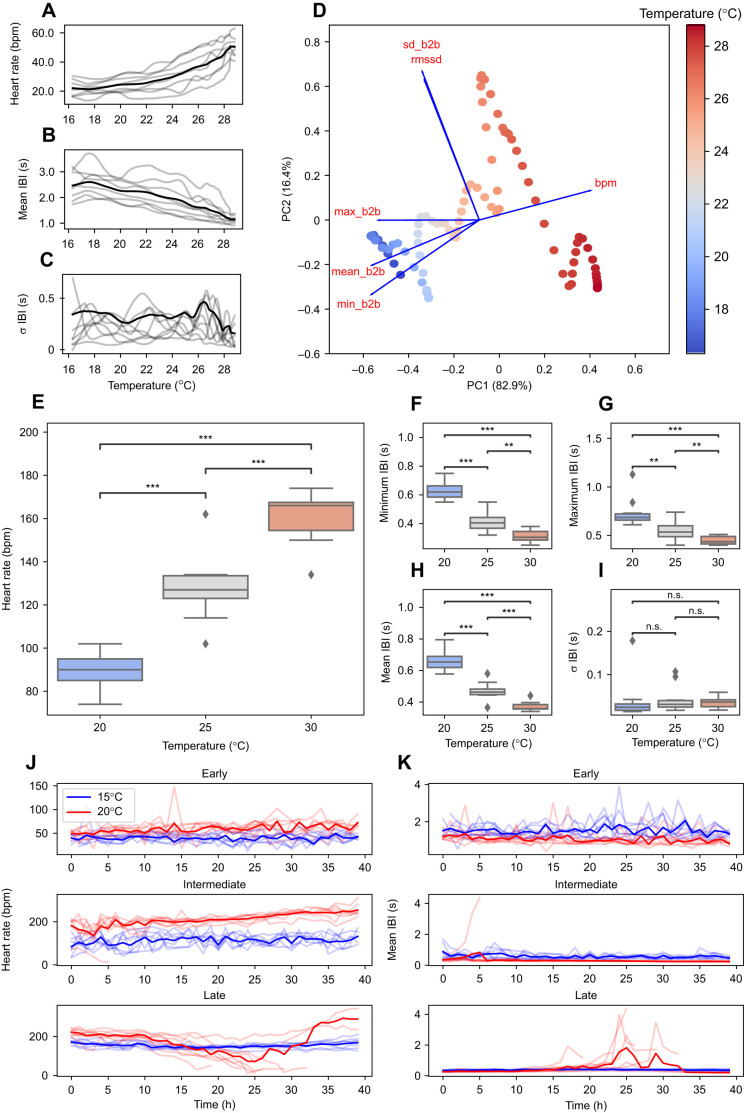
**Application of HeartCV to experiments of different design with the three species studied.** Relationship between temperature and (A) heart rate (HR), (B) mean inter-beat interval (IBI) and (C) standard deviation in IBI in 2nd ascidian stage *C. intestinalis* (no. juveniles=8). (D) The first two principal components of a PCA for HR (bpm), minimum (min_b2b), maximum (max_b2b), mean (mean_b2b), standard deviation (sd_b2b) and RMSSD (rmssd) in IBI. A bi-plot of these variables is also overlayed with the coordinate space additionally scaled by temperature. (E) HR and (F–I) IBI measures quantified for hippo stage *R. balthica* exposed to a range of constant temperatures (*n*=10 for 20°C, 25°C and 30°C). **P≤0.01, ***P≤0.005 determined via one-way ANOVA followed by Tukey's *post hoc* comparisons; n.s., non-significant results. Developmental time series for HR (J) and mean IBI (K) quantified for three different developmental stages (early, intermediate, late) of *P. serratus* in response to chronic elevated temperatures (*n*=8 per developmental stage for both 15°C and 20°C). Solid lines represent the mean response whilst translucent lines represent individual responses.

To test the applicability of HeartCV to dynamic treatments, rapid thermal ramping assays were conducted with 2nd ascidian stage *C. intestinalis* (*n*=8). Heart rate and mean IBI exhibited gradual changes with increasing temperature (Fig. 3A,B); however, standard deviation in IBI exhibited more erratic trends throughout the assays (Fig. 3C). To visualize the integrated cardiac response of *C. intestinalis* encompassing all the cardiac measures produced by HeartCV, principal component analysis (PCA) was used (Fig. 3D). The first two principal components (PC1, PC2) captured 99.4% of the variance in the cardiac measures. The combinatorial signal quantified via PCA revealed relationships between the various cardiac measures and temperature that were not apparent based on individual comparisons alone. PC1 was primarily driven by heart rate and minimum, mean and maximum IBI (Fig. 3D), whereas standard deviation and RMSSD in IBI were most aligned with PC2 (Fig. 3D), indicating that these variables are responsible for the breakpoints observed in the coordinate space. The rapid decline in standard deviation in IBI above 26°C could indicate that these individuals would experience sudden cardiac arrest if the ramping continued to higher temperatures: a consistent short-term reduction in the variation in IBIs is a key indicator for sudden cardiac arrest in humans (Schechtman et al., 1992; Stein et al., 1994; Tsuji et al., 1994; Hillebrand et al., 2013), but also in mice (Norman et al., 2012) and rainbow trout (Zena et al., 2021). Indeed, other studies measuring thermal performance in *C. intesinalis* have observed that individuals were able to tolerate static exposures of up to 28°C for 6 h (Serafini et al., 2011) and cardiac arrest was only observed above 32°C during short term static exposure (<1 h) (Wolf, 1932), so the detection of this response at a significantly lower temperature is of particular interest.

### Cardiac responses to contrasting thermal environments in hippo stage *R. balthica*

To assess the ability of HeartCV to measure cardiac responses to chronic thermal assays, we analyzed video of hippo stage *R. balthica* embryos exposed to three constant temperatures (20, 25 and 30°C). There were significant effects of temperature on HR (*F*_2,27_=36.29, *P*<0.0001), minimum (*F*_2,27_=33.97, *P*<0.0001), maximum (*F*_2,27_=16.08, *P*<0.0001) and mean IBI (*F*_2,27_=59.28, *P*<0.0001) (Fig. 3E–I), but no effect on standard deviation in IBIs (*F*_2,27_=2.957, *P*=0.901) (Fig. 3I). Whilst heart rate and mean, minimum and maximum IBI responded clearly to increasing temperature, standard deviation in IBI exhibited little change with increasing temperature (Fig. 3E–I). These responses are unsurprising given that the thermal optimum (*T*_opt_) for respiration in adults has been found to be at 33–38°C (Schaum et al., 2018), and upper critical thermal limits (CT_max_) in juveniles were identified at 36–38°C, using loss in foot attachment as an endpoint (Johansson and Laurila, 2017). Therefore, any aberrations in HR and HRV may not be observed until warmer temperatures are reached. Furthermore, development to hatching has been observed at 30°C in this population (Tills et al., 2018).

### Cardiac responses to chronic elevated temperatures in three developmental stages of *P. serratus*

Time-series video of three developmental stages of *P. serratus* exposed to chronically elevated temperatures was analyzed using HeartCV. Significant effects of temperature on the cardiac traits measured by HeartCV were evident: embryos at early and intermediate developmental stages exhibited significantly increased HR (young: *F*_1,78_=208.6, *P*<0.0001; medium: *F*_1,78_=399.5, *P*<0.0001, Fig. 3J) and consequently reduced mean IBI at 20°C relative to 15°C (young: *F*_1,78_= 165.5, *P*<0.0001; medium: *F*_1,78_=111.5, *P*<0.0001, Fig. 3K). Embryos at intermediate developmental stages exhibited more constrained mean IBI at higher temperatures (Fig. 3K). Conversely, whilst embryos at late developmental stages exhibited significantly increased mean HR (*F*_1,30_=55.28, *P*<0.0001, Fig. 3J) and reduced mean IBI (*F*_1,30_=36.5, *P*<0.0001, Fig. 3K) early on in the exposure period (0–15 h) at higher temperatures, this was followed by a collapse in heart function in most animals (*n=*6), evidenced by the marked decrease in mean HR (*F*_1,20_=13.91, *P*<0.001) and rapid increase in mean IBI parameters (*F*_1,20_=6.779, *P*<0.01) (15–25 h) (Fig. 3J–K). The gradual increase in mean HR in the remaining timepoints (25–40 h) was caused by two remaining embryos whose development continued (Fig. 3J).

Together, these responses indicate an increased sensitivity to chronic warmer temperatures in late developmental stages of *P. serratus*. In adult *P. serratus*, loss of the righting reflex has been observed at ∼33°C (Madeira et al., 2015), and so the mortality observed at 20°C here would suggest heightened embryonic thermal sensitivity. However, it should be noted that Madeira et al. (2015) acclimated adults at 20°C and the thermal assays were dynamic, in contrast to the acclimation at 15°C preceding static thermal assays in the present study. The sensitivity of critical thermal limits to methodological context is widely recognized, and a matter of considerable debate (Terblanche et al., 2007; Rezende et al., 2014; Jørgensen et al., 2019). Thus, differences in responses between that of the present study and Madeira et al. (2015) could be attributed to these methodological differences.

### Conclusion

HeartCV is an open-source Python package for noninvasive quantification of cardiac rhythm traits from video of transparent animals and encompasses an effective automated localization technique that is highly transferrable and versatile, making it a powerful tool for experimental biologists.

## Supplementary Material

10.1242/jexbio.244729_sup1 Supplementary information
