# Supplementary material for: HeartCV: a tool for transferrable, automated measurement of heart rate and heart rate variability in transparent animals
Source: J Exp Biol. 2022 Oct 3;225(19):jeb244729. doi: 10.1242/jeb.244729 (PMC9659326; doi:10.1242/jeb.244729)
Supplement: Supplementary information [file jexbio-225-244729-s1.pdf]

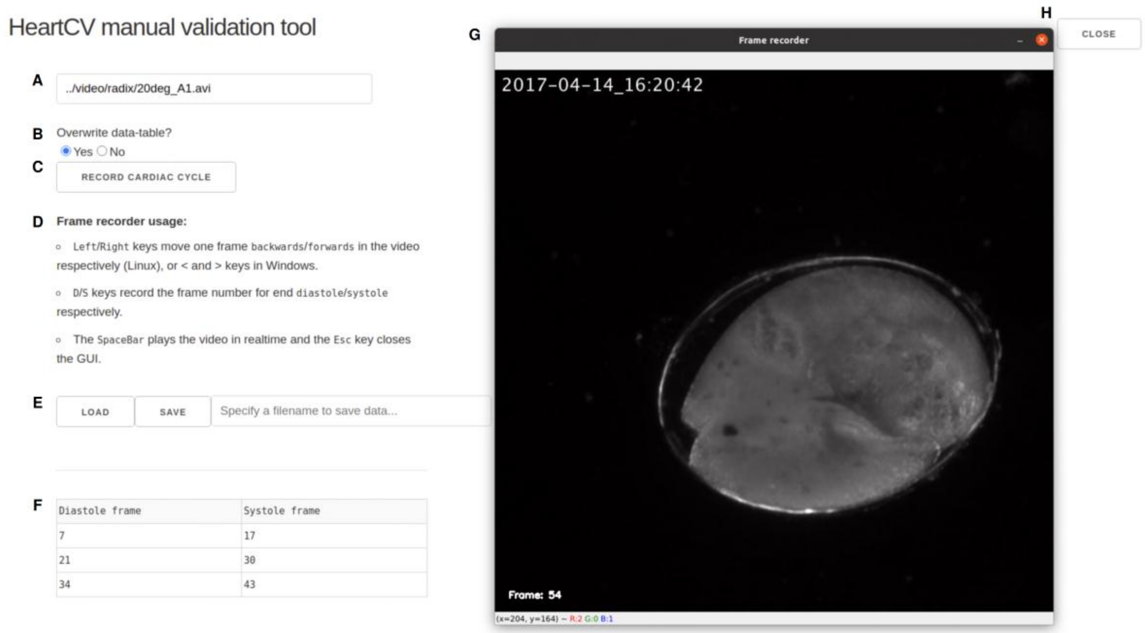


**Fig. S1.** User interface for manual quantification of heart rate and IBI measures for comparison with measures derived from automated quantification. Users can specify individual videos to validate via a file input (A) and whether over-write existing data through the use of toggle buttons (e.g. when validating multiple videos in one session) (B). The frames at which a given cardiac event occurs can be recorded through the use of an interactive window (C and G) which is responsive to keyboard presses (D). These results are then stored in a table (F) and can be exported to CSV format (E).
